# Markers of Inflammation and Monoamine Metabolism Indicate Accelerated Aging in Bipolar Disorder

**DOI:** 10.3389/fpsyt.2018.00250

**Published:** 2018-06-14

**Authors:** Seline van den Ameele, Dietmar Fuchs, Violette Coppens, Peter de Boer, Maarten Timmers, Bernard Sabbe, Manuel Morrens

**Affiliations:** ^1^Faculty of Medicine and Health Sciences, University of Antwerp, Antwerp, Belgium; ^2^University Psychiatric Hospital Duffel - VZW Emmaüs, Duffel, Belgium; ^3^Division of Biological Chemistry, Biocenter, Medical University of Innsbruck, Innsbruck, Austria; ^4^Janssen Research and Development, A Division of Janssen Pharmaceutica N.V., Beerse, Belgium; ^5^Reference Center for Biological Markers of Dementia, Institute Born-Bunge, University of Antwerp, Antwerp, Belgium

**Keywords:** bipolar disorder, inflammation, monoamines, neopterin, neuroprogression, accelerated aging

## Abstract

**Background:** A mild pro-inflammatory status accompanies bipolar disorder (BD). Inflammation can cause a shift in monoamine metabolism, thereby activating more cytotoxic pathways. The extent to which low-grade inflammation in BD interacts with monoamine metabolism and how this accords to aging and clinical course is unknown.

**Objectives:** We evaluated the presence of alterations in inflammation and monoamine metabolism in BD throughout different mood states and the role of aging therein.

**Methods:** Sixty-seven patients with BD were included during an acute mood episode, either depressive (*n* = 29), (hypo)manic (*n* = 29), or mixed (*n* = 9). Plasma levels of inflammatory markers [tumor necrosis factor alpha (TNF-α), interferon gamma (IFN-y), interleukin-6 (IL-6), and C-reactive protein (CRP)] and markers of monoamine metabolism (neopterin, tryptophan, kynurenine, phenylalanine, and tyrosine) were measured repeatedly during a follow-up of 8 months. Levels in patients were compared to controls (*n* = 35) and correlated to HDRS-17 and YMRS scores. Spearman correlations and linear mixed model analysis were used for statistical analysis.

**Results:** Forty-nine patients and 30 controls (age range: 22–62 years) completed the study. No significant differences in inflammatory markers were found between patients and controls overall. Tryptophan, tyrosine, and phenylalanine levels were lower in patients. In both patients and controls, markers of inflammation correlated only weakly with markers of monoamine metabolism, but correlations representative for activity of cytotoxic pathways in monoamine metabolism were more pronounced in patients. In patients, but not in controls, older age was associated with increases in inflammatory markers (IL-6, CRP, neopterin) and the kynurenine/tryptophan ratio. None of the biological markers correlated significantly with mood symptom severity.

**Conclusion:** Our data suggest an increased susceptibility of patients with BD to develop a pro-inflammatory state and to shift monoamine metabolism toward more cytotoxic pathways. These findings are in support of the theory of neuroprogression and accelerated aging in BD. Since associations between biological markers and clinical characteristics are limited, it remains to be determined if alterations in biological markers are due to a disease effect or rather are a consequence of confounding factors.

## Introduction

Low-grade inflammation has been documented extensively in bipolar disorder (BD). Increased levels of pro-inflammatory cytokines and acute phase proteins have been shown during acute mood episodes (1–5) and in later stages of the disease (4, 6). Even during euthymia, isolated monocytes were found to express more pro-inflammatory genes, and the activity of hippocampal microglia was increased (7–9). According to the theory of accelerated aging, the early onset of a chronic low-grade inflammation underlies neuroprogression in BD by affecting monoamine synthesis and increasing the production of cytotoxic metabolites (10, 11).

**Figure 1:**
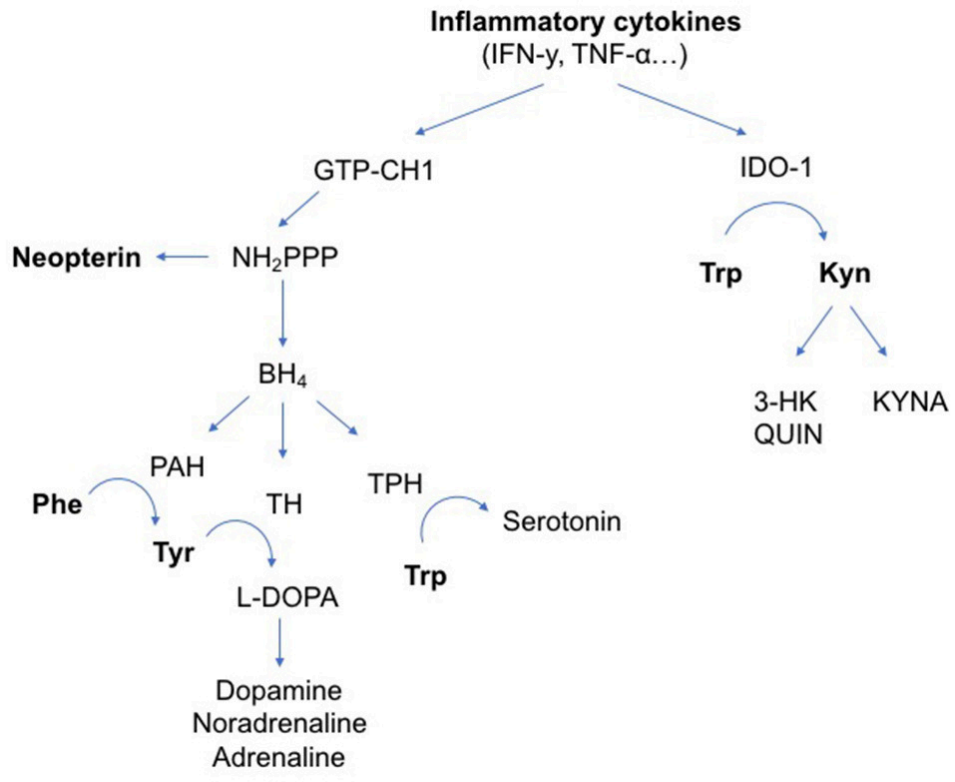
Influence of inflammatory cytokines on neurotransmitter metabolism. The pro-inflammatory cytokines IFN-y and TNF-α can activate enzymatic pathways that change neurotransmitter metabolism. IFN-y stimulates GTP-CH1, resulting in the synthesis of BH_4_ and neopterin. TNF-α further enhances IFN-y-stimulated GTP-CH1 activity. Neopterin is almost exclusively synthesized in and released by activated cells of the monocyte/macrophage system and used as a marker for activated cell-mediated immunity. Increased neopterin levels also indicate increased oxidative stress induced by immune-mediated processes. In chronic inflammation, mainly neopterin is released at the expense of BH_4_ production. BH_4_ is an essential cofactor in the synthesis of dopamine, noradrenaline, adrenaline and serotonin. IFN-y and TNF-α also stimulate IDO-1 activity. Upon immune activation, IDO-1 converts Trp to Kyn and thus depletes Trp for serotonin synthesis. Downstream kynurenine metabolites have several cytotoxic or neuroactive effects. Arg, L-Arginine; BH_4_, tetrahydrobiopterin; GTP-CH1, guanosine triphosphate cyclohydroxylase 1; IDO-1, indoleamine 2,3 dioxygenase 1; IFN-y, interferon gamma; IL, interleukin; iNOS, inducible nitric oxide synthase; Kyn, kynurenine; KYNA, kynurenic acid; L-DOPA, L-3,4-dihydroxyphenylalanine; NH_2_*PPP*, dihydroneopterin triphosphate; NO, nitric oxide; PAH, phenylalanine hydroxylase; Phe, phenylalanine; TH, tyrosine hydroxylase; TNF-α, tumor necrosis factor alpha; TPH, tryptophan hydroxylase; Trp, tryptophan; Tyr, tyrosine; QUIN, quinolinic acid; 3-HK, 3-hydroxykynurenine.

Rising evidence suggests that immune system pathways act on monoamine biosynthesis (See Figure 1) (12–14). In chronic inflammation, activation of guanosine triphosphate cyclohydroxylase 1 (GTP-CH1) by the pro-inflammatory cytokines interferon gamma (IFN-y) and tumor necrosis factor-alfa (TNF-α) results in increased neopterin production at the expense of tetrahydrobiopterin (BH_4_). Neopterin is a marker of activated cell-mediated immunity and increased oxidative stress (15, 16), while BH_4_ is an essential cofactor in the synthesis of dopamine, noradrenaline, adrenaline and serotonin (17–19). IFN-y and TNF-α also stimulate indoleamine 2,3 dioxygenase 1 (IDO-1) activity. Upon immune activation, IDO-1 converts tryptophan to kynurenine and thus depletes tryptophan for serotonin synthesis. Kynurenine metabolites have several downstream cytotoxic or neuroactive effects (14, 20).

In viral infections, cancer and autoimmune diseases, inflammatory markers have already been correlated with monoamine synthesis and neopterin production (16). As seen in healthy aging, low-grade inflammation correlates to increased neopterin levels and IDO-1-mediated induction of tryptophan metabolism. In otherwise healthy elderly IDO-1 and GTP-CH1 activity have been associated with depressive symptomatology (17). Decreased levels of BH_4_ have been found in patients with major depression and schizophrenia (21, 22). Although changes in neopterin levels and IDO-1 activity were found in patients with BD (23, 24), it remains unclear whether these changes in monoamine synthesis correlate with inflammatory alterations.

The aim of this study was to evaluate a possible association between inflammation and monoamine metabolism in bipolar disorder and its relation to aging and clinical course. We hypothesized pro-inflammatory cytokines to be increased in patients with BD compared to healthy controls, and more so during mood episodes and in older patients with a longer duration of illness, resulting in an activation of GTP-CH1 and IDO-1.

## Methods

### Participants

Inpatients were recruited in 3 psychiatric centers in the region of Antwerp, Belgium. Outpatients were recruited via the Flemish patient association. The inclusion criteria were age 18–65 years, DSM-IV diagnosis of BD type I, type II or schizoaffective disorder and suffering from a depressive or (hypo)manic episode at time of inclusion. Clinical assessments are described below (see section Clinical Assessments). Age and gender matched controls were recruited mainly among staff members of the participating centers. We ensured an equal distribution of inclusions of patients and controls throughout the year to account for seasonality in immune system activity (25). Exclusion criteria for both patient and control group were: substance abuse, use of anti-inflammatory drugs within 2 weeks preceding screening or test days, acute infection, autoimmune diseases, chronic inflammatory or neurological diseases, pregnancy or breastfeeding, electroconvulsive therapy (ECT) within 6 months before screening or during follow-up, mental retardation, significant disturbances on a screening blood test evaluating complete blood count, electrolytes, fasting glucose, lipid profile, liver, kidney and thyroid function, and serology (human immunodeficiency virus, hepatitis B and C). Urine drug testing was routinely done at screening and repeated on subsequent test days when drug abuse was suspected (e.g., history of substance abuse, unreliable anamnesis). In the control group, additional exclusion criteria were applied: current or past diagnosis of major depressive disorder, BD or psychotic syndrome as defined by DSM-IV criteria and BD or psychotic syndrome in a first-degree family member and current use of psychopharmacological drugs. There were no other restrictions regarding medication use.

Participants were recruited between March 2015 and May 2016. The study was approved by the Committee for Medical Ethics of the University Hospital Antwerp and the Antwerp University with protocol number B300201421645. The local ethical committees of the participating centers approved the protocol. All participants agreed to participate in the study and signed informed consent. The study complied with the Declaration of Helsinki.

### Study design

Patients were recruited during an acute mood episode, either depressed, (hypo)manic or mixed. In both patients and controls, screening was followed by a first test day after 1–5 days. Subsequent test days were planned after, respectively 1, 2, 4, 6, and 8 months of follow-up, resulting in 6 test days per participant over the course of 8 months. Every test day included the same clinical and laboratory assessments as described below. During the study period, patients received treatment as usual without intervention of the investigators.

### Clinical assessments

The M.I.N.I.-plus, International Neuropsychiatric Interview, version 5.0.0 was chosen as diagnostic instrument in patients and controls because of its accurate structured DSM-IV diagnosis and convenience to administer (26). In patients, the severity of mood symptoms was assessed by the 17-item Hamilton Depression Rating Scaling (HDRS-17) (27) and the Young Mania Rating Scale (YMRS) (28) at screening and on all test days. At screening, threshold score for inclusion was set at ≥17 for the HDRS-17 or ≥13 for the YMRS, corresponding to moderate depression or hypomania, respectively (29, 30). On all subsequent test days, the mood state of patients was classified as “depressive,” “(hypo)manic,” “mixed,” or “euthymic” according to the HDRS and YMRS scores. Psychotic symptoms were evaluated on test days using the positive subscale of the Positive and Negative Syndrome Scale (PANSS) (31). In the control group, the occurrence of mood episodes during follow-up was evaluated on all test days based on a short screening questionnaire. For all participants, we assessed medication use and the occurrence of any of the exclusion criteria on every test day. All clinical assessments were done by a psychiatrist in training (SvdA) and supervised by a psychiatrist (MM).

### Laboratory assessments

Blood was drawn by venipuncture between 08.00 and 10.30 a.m. into a citrate vacuum tube (2.7 ml). Tubes were immediately stored at 4°C, centrifuged at 2 g and 4°C for 10 min within 2 h after blood draw, and plasma was aliquoted and stored at −70°C until assayed.

TNF-α, IFN-y, IL-1β, IL-4, IL-6, and CRP were measured in duplicate by an electrochemiluminescence immunoassay technique developed by Mesoscale Discovery (Rockville, USA) according to the manufacturer's instructions. Kits used for detection were V-plex Pro-inflammatory Panel I for TNF-α, IFN-y, IL-1β, IL-4, and IL-6 and Human Vascular Injury Panel for CRP. The lower limits of detection (LLOD) were, respectively 0.04, 0.2, 0.04, 0.02, 0.06, and 1.3 pg/ml. Sample signals were fitted on a 4-parametric logistic calibration curve to calculate concentrations.

Patient and control samples were analyzed in randomized sequence with both samples of a single subject on the same plate and an equal distribution of patients and controls per plate. Samples with a coefficient of variation (CV) >20% were excluded from statistical analyses. Because >80% of the samples were below the LLOD for IL-1β and IL-4, these cytokines were not included in statistical analyses. Excluded samples were equally distributed among patients and controls. For TNF-α, IFN-y, IL-6, and CRP, >80% of the samples were included for analysis. The mean CV and standard deviation were 6.3 (4.7), 8.4 (5.5), 7.6 (5.5), 5.4 (4.5), respectively.

Neopterin concentrations were determined by enzyme-linked immunosorbent assay according to the manufacturer's instructions (BRAHMS Diagnostics, Hennigsdorf, Germany). Tryptophan, kynurenine, phenylalanine, and tyrosine were determined by high-performance liquid chromatography, as described previously (32, 33). The ratios of Kyn/Trp and Phe/Tyr were calculated as indexes of IDO-1 and PHA activity, respectively. The Phe/Tyr ratio is also used as a reliable measure of BH_4_ availability (34).

### Statistical analysis

Normality of outcome variables and homoscedasticity of residuals were evaluated by visual inspection. For the regression modeling, IFN-y, IL-6, TNF-α, and CRP concentrations were log-transformed to obtain a normal distribution. Homogeneity of variances was assessed by Levene's test and further analyses were adapted accordingly.

Baseline differences in clinical and demographic parameters between the patient and control group were examined by two-tailed independent *t-*tests for continuous variables and Pearson chi-square test for categorical variables.

Longitudinal data were examined using linear mixed model analysis with the biological parameters as outcome variable. Based on the *LogLikelihood* value, we fitted a model that included the subject ID as random intercept. We included subsequently group (patient vs. HC) and mood state [depression vs. (hypo)mania vs. mixed episode vs. euthymia vs. controls] as fixed effects. Smoking status and BMI were added as covariates to the adjusted models. As nutritional status affects amino acid levels, albumin concentrations were also added as covariates in the adjusted model of amino acid level prediction. Impact of age was assessed by adding age and the interaction between age and group as covariates in the linear mixed model. The output from the mixed model analysis, is reported as “F-ratio (DF); *p*-value; *b*.”

The relation among biological parameters and the relation between symptom severity scores and biological parameter levels were studied by pairwise correlations. Correlations are reported by the Spearman's rho for non-parametric distributions. *P-*values below 0.05 were considered statistically significant. All statistical analyses were performed in JMP Pro 12 (JMP, Marlow, UK).

## Results

### Participants

Sixty-seven patients with BD and 35 controls were included. At screening, 29 patients had a depressive episode, 29 patients a hypomanic or manic episode, and 9 patients a mixed episode. Demographic and metabolic characteristics are shown in Table 1. Patients and controls were matched by sex and age. Body mass index (BMI) and the percentage of smokers were higher in patients compared to controls. No other significant differences in demographic or metabolic parameters were found between patients and controls. Clinical characteristics of patients are shown in Table 2.

**Table 1:** Baseline demographic and metabolic characteristics.

	**Patients**	**Controls**	***p*-value^a^**
N	67	35	
Gender, female	39 (58.2)	19 (54.3)	0.704
Age, years	43.3 ± 11.1 (23–62)	42.7 ± 11.6 (23–62)	0.883
Caucasian	63 (94.0)	34 (97.1)	0.489
Smokers	32 (47.8)	6 (17.1)	**0.002**
BMI, kg/cm^2^	25.3 ± 4.2 (18–39)	23.7 ± 2.6 (20–29)	**0.025**
Waist, cm	89.1 ± 12.1 (66–122)	84.4 ± 9.6 (67–104)	0.059
Fasting glucose, mg/dl	90.0 ± 9.3 (69–116)	87.5 ± 6.8 (73–102)	0.176
Cholesterol, mg/dl			
Total	186.5 ± 44.7 (101–341)	190.9 ± 42.4 (132–283)	0.636
HDL	58.6 ± 17.8 (24–102)	62.0 ± 18.4 (28–118)	0.372
LDL	105.6 ± 40.9 (44–264)	109.0 ± 33.4 (57–184)	0.674

Data presented as mean ± SD (range) or n (%). BMI, body mass index; HDL, high density lipoprotein; LDL, low density lipoprotein.

a *p-values of t-test or Chi-squared test. Bold values: significant p-values (p < 0.05)*.

**Table 2:** Clinical characteristics and baseline data of patients.

N	67
Diagnosis	
BD type I	42 (62.7)
BD type II	23 (34.3)
Schizoaffective disorder	2 (3)
Age of onset, years	24.9 ± 11.5 (8-55)
Duration of illness, years	17.6 ± 11.3 (0-49)
First episode: depression	40 (60.6)
Age first depression, years	25.9 ± 12.1 (8-55)
Age first mania/hypomania, years	28.9 ± 11.9 (8-59)
Lifetime psychotic features	37 (55.2)
Total number of hospitalizations	
0	9 (13.4)
1-5	45 (67.2)
6-10	13 (19.4)
Lifetime substance abuse	30 (44.8)
Alcohol	18 (26.9)
THC	13 (19.4)
Hard drugs	5 (7.5)
Baseline medication use	
Medication-free	6 (9.0)
Lithium	24 (35.8)
Valproate	9 (13.4)
Carbamazepine	3 (4.5)
Lamotrigine	8 (11.9)
Antipsychotic	42 (62.7)
Antidepressant	31 (46.3)
Benzodiazepine	24 (35.8)
Baseline mood episode	
Depression	29 (43.3)
(Hypo)mania	29 (43.3)
Mixed	9 (13.4)

*Data presented as mean ± SD (range) or n (%). BD, bipolar disorder; THC, tetrahydrocannabinol*.

Forty-nine patients and 30 controls completed the 8-month's study design. Drop-out in the patient group was due to: chronic use of low-dose acetylsalicylic acid (*n* = 5), substance abuse (*n* = 3), ECT (*n* = 2), and loss of contact or lack of motivation for further participation (*n* = 8). Drop-out in the control group was due to difficult blood draws (*n* = 1) and repeated orthopedic surgery (*n* = 1). Three controls were only included for baseline testing. The mean number of test days per participant was 5.0 in patients and 5.4 in controls. Over 8 months, the total number of blood samples included for analyses was 336 in patients and 188 in controls. In total, 178 test moments were during a depressive episode, 71 during a (hypo)manic episode, 23 during a mixed episode, and 64 test moments were during a euthymic episode. Symptom severity scores by mood state are shown in Table 3 and Table S1.

**Table 3:** Mood symptom severity and differences in biological markers between mood states and controls.

	**Depression**	**(Hypo)mania**	**Mixed**	**Euthymia**	**Controls**	**Effect**	**Tukey HSD**
# Test moments (N)	178	71	23	64	188		
HDRS	16.6 (5.8)	6.6 (4.1)	20.5 (4.4)	3.9 (2.2)			
YMRS	3.5 (2.7)	18.3 (7.0)	15.2 (4.4)	2.7 (2.4)			
IFN-y (pg/ml)^*^	4.24 (0.08)	4.67 (0.10)	4.71 (0.15)	4.78 (0.10)	4.87 (0.10)	*F*_(403.4)_ = 0.6; *p* = 0.655	
IL-6 (pg/ml)^*^	0.49 (0.07)	0.52 (0.08)	0.42 (0.12)	0.52 (0.08)	0.41 (0.09)	*F*_(434)_ = 1.6; *p* = 0.168	
TNF-α (pg/ml)^*^	1.95 (0.04)	1.89 (0.04)	1.83 (0.06)	1.87 (0.04)	1.77 (0.05)	*F*_(498.4)_ = 1.5; *p* = 0.198	
CRP (mg/L)^*^	1.81 (0.15)	2.08 (0.15)	2.16 (0.21)	1.97 (0.15)	1.27 (0.17)	*F*_(487.7)_ = 1.5; *p* = 0.205	
Trp (μmol/l)	50.04 (0.99)	51.67 (1.24)	54.61 (2.02)	51.29 (1.28)	54.37 (1.18)	*F*_(333.2)_ = 2.8; ***p*** = **0.025**	D < C
Kyn (μmol/l)	1.43 (0.04)	1.46 (0.05)	1.53 (0.08)	1.51 (0.05)	1.58 (0.05)	*F*_(350.7)_ = 1.9; *p* = 0.116	
Kyn/Trp (μmol/mmol)	28.89 (0.75)	28.89 (0.91)	28.54 (1.42)	29.98 (0.93)	29.41 (0.92)	*F*_(351.5)_ = 0.5; *p* = 0.702	
Neo (nmol/l)	4.96 (0.17)	5.00 (0.23)	5.12 (0.40)	5.44 (0.25)	5.04 (0.18)	*F*_(306.1)_ = 0.8; *p* = 0.499	
Tyr (μmol/l)	62.64 (2.25)	70.62 (2.78)	71.67 (4.45)	64.10 (2.87)	75.06 (2.71)	*F*_(345.3)_ = 4.9; ***p*** < **0.001**	D < C & M; E < C
Phe (μmol/l)	49.47 (1.12)	52.08 (1.43)	56.13 (2.35)	50.54 (1.48)	55.93 (1.31)	*F*_(334.7)_ = 4.9; ***p*** < **0.001**	D < C & Mx
Phe/Tyr	0.83 (0.02)	0.77 (0.22)	0.81 (0.03)	0.82 (0.02)	0.78 (0.02)	*F*_(346.3)_ = 2.4; *p* = 0.053	

Data presented as mean (SE). SE, standard error; HDRS, Hamilton depression rating scale; YMRS, Young mania rating scale; IFN-y, interferon gamma; IL, interleukin; TNF-α, tumor necrosis factor alpha; CRP, C-reactive protein; Trp, tryptophan; Kyn, kynurenine; Neo, neopterin; Tyr, tyrosine; Phe, phenylalanine; D, depression; C, controls; E, euthymia; M, (hypo)mania; Mx, mixed.

* *SE on log-transformed data. Bold values: significant p-values (p < 0.05)*.

### Biological markers in patients and healthy controls

#### Biological markers in patients vs. controls

The overall levels of inflammatory markers and amino acids in patients and controls are shown in Table S2. No significant differences in levels of inflammatory markers (IFN-y, IL-6, TNF-α, and CRP) were found between patients and controls. Patients had significantly lower levels of tryptophan [*F*_(92.4)_ = 5.2; *p* = 0.026; *b* = 3.37], tyrosine [*F*_(98.2)_ = 7.8; *p* = 0.006; *b* = 9.56], and phenylalanine [*F*_(95.0)_ = 9.6; *p* = 0.003; *b* = 5.14] as compared to controls. After adjustment for BMI and albumin levels, also kynurenine was found to be lower in patients [*F*_(95.6)_ = 3.9; *p* = 0.0499; *b* = 0.13]. After adjustment for smoking status, kynurenine remained significantly lower in patients [*F*_(99)_ = 4.0; *p* = 0.048; *b* = 0.16]. Neopterin and the Phe/Tyr and Kyn/Trp ratios did not differ significantly between patients and controls.

Differences between controls and different mood states in patients [i.e., depression vs. (hypo)mania vs. mixed episode vs. euthymia vs. controls] are shown in Table 3. No differences in levels of inflammatory markers were found. The decrease in tryptophan, tyrosine, and phenylalanine found in the total patient group is significantly more pronounced in depressive patients. These findings did not retain statistical significance after adjustment for smoking status.

#### Correlation between markers of inflammation and amino acids in patients vs. controls

In both patients and controls, inflammatory parameters correlated positively to neopterin levels. IFN-y and TNF-α positively correlated to Kyn/Trp in both groups. In patients, but not in controls, IL-6 correlated positively to the Kyn/Trp ratio and negatively to tryptophan. Neopterin correlated positively to kynurenine and the Kyn/Trp and Phe/Tyr ratios, with a stronger correlation in patients. Further details on correlations are shown in Table 4 and Table S3.

**Table 4:** Spearman correlations between markers of inflammation and monoamine metabolism in patients vs. controls.

	**Neopterin**		**Kyn/Trp**		**Phe/Tyr**	
	**Patients**	**Controls**	**Patients**	**Controls**	**Patients**	**Controls**
IFN-y	0.31^***^	0.35^***^	0.17^**^	0.34^***^	0.05	−0.11
IL-6	0.20^**^	0.28^**^	0.19^**^	0.15	−0.14^*^	−0.06
TNF-α	0.33^***^	0.26^***^	0.32^***^	0.27^*^	0.04	−0.04
CRP	0.23^***^	0.25^***^	−0.01	0.1	−0.04	−0.05
Neopterin	1	1	0.43^***^	0.27^***^	0.17^**^	−0.07

* p < 0.05;

** p < 0.01;

*** *p < 0.001. Trp, tryptophan; Kyn, kynurenine; Tyr, tyrosine; Phe, phenylalanine; IFN-y, interferon gamma; IL, interleukin; TNF-α, tumor necrosis factor alpha; CRP, C-reactive protein*.

#### Impact of age and course of illness on biological markers

Correlations between inflammatory markers and symptom severity scores were weak and not significant (see Table S4). Small inverse correlations were found between HDRS scores and tryptophan (ρ = −0.13), kynurenine (ρ = −0.14), and tyrosine (ρ = −0.11) levels, while positive correlations (ρ between 0.15 and 0.22) were found between YMRS and PANSS positive subscale scores and tryptophan, kynurenine, tyrosine, and phenylalanine levels. Neopterin levels and the Phe/Tyr and Kyn/Trp ratios were not significantly correlated to symptom severity scores, except for a weak positive correlation between Phe/Tyr and HDRS scores (ρ = 0.13). See Table S4 for further details. In line with the correlations, the presence of psychotic features was associated with an increase in phenylalanine levels [*F*_(318.4)_ = 9.1; *p* = 0.003; *b* = 4.99].

**Figure 2:**
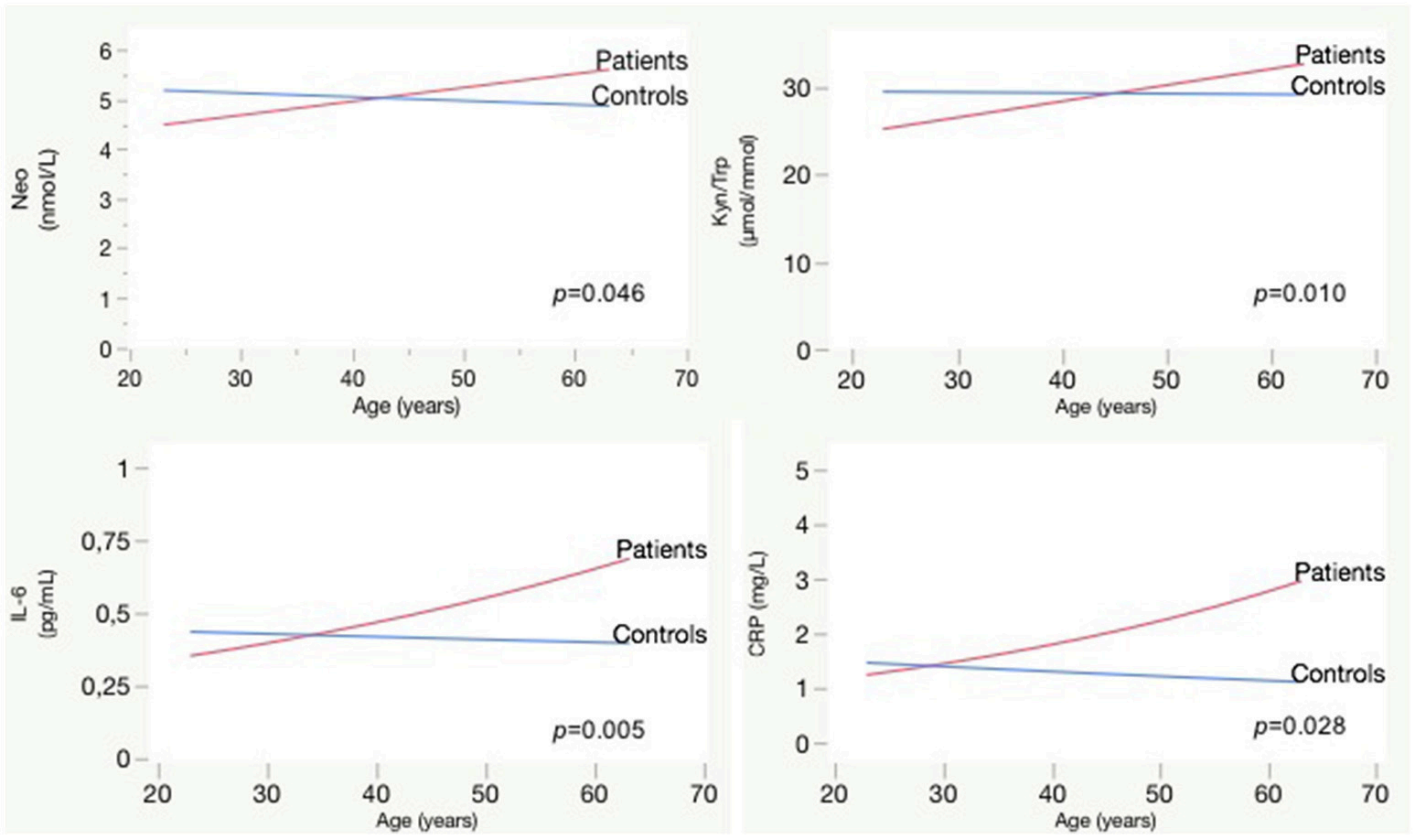
Effect of aging on Neopterin, Kyn/Trp, IL-6, and CRP in patients vs. controls: interaction plots. CRP, C-reactive protein; IL, interleukin; Kyn, kynurenine; Neo, neopterin; Trp, tryptophan.

Longer duration of illness was associated with higher TNF-α [*F*_(65.2)_ = 5.3; *p* = 0.024; *b* = 0.01], kynurenine [*F*_(64.7)_ = 4.3; *p* = 0.042; *b* = 0.01], Kyn/Trp [*F*_(66.4)_ = 8.0; *p* = 0.006; *b* = 0.17], and neopterin [*F*_(70.2)_ = 6.2; *p* = 0.015; *b* = 0.03], effect sizes are rather small. Comparing the impact of aging on biological parameters between patients and controls, we observed a significant interaction effect between age and patient/control status for IL-6, CRP, neopterin, and the Kyn/Trp ratio. Patients have rising IL-6, CRP, neopterin, and Kyn/Trp ratio with older age, while these parameters are stable in controls (see Figure 2). A similar age effect is found when comparing biomarker levels in participants below and above 45 years of age (see Table 5). Only in the patient group we observed a significant difference between both age groups: the older patient group had higher levels of Kyn/Trp, neopterin, IL-6, and TNF-α and lower tryptophan levels. Comparing patients and controls, the <45 years group had lower levels of kynurenine, Kyn/Trp, and tyrosine in patients (*p* = 0.012, 0.018, and 0.040, respectively), while inflammatory markers were not different between patients and controls. Patients in the >45 years group had lower levels of tryptophan and phenylalanine (*p*-value of 0.009 and 0.006, respectively) compared to controls and higher IL-6, TNF-α, and CRP levels (*p*-values of 0.003, 0.047, and 0.007).

**Table 5:** Differences in biological markers in patients and controls below and above 45 years of age.

	**Patients**			**Controls**		
	**<45 y (*n* = 36)**	**≥45 y (*n* = 31)**	**Effect**	**<45 y (*n* = 19)**	**≥45 y (*n* = 16)**	**Effect**
Trp (μmol/l)	52.91 (1.35)	48.70 (1.8)	*F*_(61.6)_ = 5.3; ***p*** = **0.025**; *b* = 4.21	53.81 (1.30)	55.15 (1.34)	*F*_(150)_ = 0.8; *p* = 0.369; *b* = −1.34
Kyn (μmol/l)	1.43 (0.05)	1.51 (0.06)	*F*_(63.5)_ = 1.0; *p* = 0.311; *b* = −0.08	1.57 (0.05)	1.59 (0.05)	*F*_(181)_ = 0.1; *p* = 0.700; *b* = −0.02
Kyn/Trp (μmol/mmol)	27.20 (0.91)	31.30 (0.99)	*F*_(65.0)_ = 9.4; ***p*** = **0.003**; *b* = −4.09	29.71 (0.87)	29.08 (0.90)	*F*_(180.5)_ = 0.5; *p* = 0.468; *b* = 0.64
Neo (nmol/l)	4.78 (0.18)	5.41 (0.20)	*F*_(67.5)_ = 5.5; ***p*** = **0.022**; *b* = −0.63	5.15 (0.22)	4.94 (0.23)	*F*_(88.8)_ = 0.6; *p* = 0.457; *b* = 0.22
Tyr (μmol/l)	64.05 (2.50)	66.34 (2.69)	*F*_(60.6)_ = 0.5; *p* = 0.498; *b* = −2.28	76.70 (4.00)	73.12 (4.14)	*F*_(179.7)_ = 0.8; *p* = 0.378; *b* = 3.52
Phe (μmol/l)	50.97 (1.26)	50.27 (1.37)	*F*_(58.2)_ = 0.2; *p* = 0.695; *b* = 0.69	55.89 (1.85)	55.88 (1.92)	*F*_(169.7)_ = 0.0; *p* = 0.998; *b* = 0.01
Phe/Tyr	0.83 (0.02)	0.79 (0.02)	*F*_(64.8)_ = 1.3; *p* = 0.250; *b* = 0.04	0.77 (0.02)	0.78 (0.03)	*F*_(160.7)_ = 0.1; *p* = 0.757; *b* = −0.01
IFN-y (pg/ml)^*^	4.66 (0.10)	4.94 (0.09)	*F*_(56.7)_ = 0.2; *p* = 0.683; *b* = −0.06	5.55 (0.13)	4.29 (0.14)	*F*_(95.5)_ = 2.0; *p* = 0.161; *b* = 0.26
IL-6 (pg/ml)^*^	0.41 (0.08)	0.61 (0.08)	*F*_(62.8)_ = 12.4; ***p*** <**0.001**; *b* = −0.41	0.42 (0.12)	0.41 (0.12)	*F*_(136.3)_ = 0.1; *p* = 0.774; *b* = 0.04
TNF-α (pg/ml)^*^	1.77 (0.05)	2.08 (0.06)	*F*_(65.2)_ = 4.4; ***p*** = **0.039**; *b* = −0.16	1.74 (0.06)	1.81 (0.06)	*F*_(174.7)_ = 0.8; *p* = 0.378; *b* = −0.04
CRP (mg/L)^*^	1.65 (0.19)	2.29 (0.20)	*F*_(67.7)_ = 1.4; *p* = 0.236; *b* = −0.33	1.41 (0.16)	1.14 (0.16)	*F*_(154.2)_ = 1.3; *p* = 0.260; *b* = 0.21

*Data presented as mean (SE)*.

SE, standard error; IFN-y, interferon gamma; IL, interleukin; TNF-α, tumor necrosis factor alpha; CRP, C-reactive protein; Trp, tryptophan; Kyn, kynurenine; Neo, neopterin; Tyr, tyrosine; Phe, phenylalanine.

* *SE on log-transformed d. Bold values: significant p-values (p < 0.05)*.

Other illness characteristics such as duration of current mood state, lifetime psychotic features, BD type I or II, number of mood episodes and number of hospitalizations revealed no significant relations with any of the biological markers (*p* > 0.05).

## Discussion

In this study we measured markers of monoamine synthesis and immune activity in patients with BD and controls. We found decreased levels of tryptophan, phenylalanine and tyrosine in patients, which were more pronounced during depressive episodes. We found no differences in inflammatory markers between the overall groups of patients vs. controls. Nonetheless, our results suggest a proneness of patients with BD for an increased pro-inflammatory state and its related cytotoxic effects as (i) correlations between inflammatory markers and monoamine metabolites diverge distinctly between patients and controls and (ii) a premature pro-inflammatory status arises in middle-aged patients and increases over the course of illness.

The positive correlation between Phe/Tyr and neopterin levels found in patients with BD is similar to the changes seen during chronic inflammation in cancer, HIV, and autoimmune diseases which are related to activation of the GTP-CH1 enzyme (18). Activation of GTP-CH1 results in increased neopterin synthesis in macrophages at the expense of BH_4_, an essential cofactor for monoamine synthesis (17). We found that IL-6 levels correlated positively to Kyn/Trp and negatively to tryptophan only in patients, which suggests activation of the IDO-1 enzyme. Increased IDO-1 activity results in higher tryptophan breakdown in the kynurenine pathway. The consequence is a lower tryptophan availability for serotonin synthesis and increased levels of kynurenine metabolites that have multiple cytotoxic and neuroactive effects. These different interactions between markers of inflammation and monoamine metabolism in patients vs. controls are suggestive for a stronger interaction between inflammation, activation of IDO-1 and GTP-CH1, impaired monoamine synthesis and increased production of cytotoxic metabolites in patients.

Interestingly, exclusively in the patient group, aging and increased duration of illness were associated with a rise in levels of pro-inflammatory markers, neopterin and the Kyn/Trp ratio. A pro-inflammatory status accompanies normal aging and is thought to underlie the increased frailty and vulnerability for psychiatric disorders in elderly (35–37). Similar to the correlations found in our patient group, chronic low-grade inflammation in healthy elderly was related to increased Phe/Tyr, and to decreased tryptophan levels (17). As in our patient group, older age was also related to increasing IL-6, neopterin, and IDO-1 activation. The mean age of 79.9 years in the above study of Capuron et al. (17) contrasts with the mean age of 43 years in our study population. Previous research shows that in healthy subjects over 60 years of age the effects of aging on inflammation and IDO-1 activation become more apparent (38, 39). Our study revealed increased pro-inflammatory markers in patients with BD above 45 years of age. Similarly, Drexhage et al. (40) demonstrated a higher proportion of regulatory T-cells in patients below 40 years, compared to controls. Regulatory T-cells temper the inflammatory response and maintain immune homeostasis and tolerance.

Both the stronger correlation between inflammation and GTP-CH1 and IDO-1 activation and the premature shift toward a pro-inflammatory status in our patient group strengthens the hypothesis of BD as a disease of accelerated aging (11). Due to both acute and chronic stress throughout the course of illness, the compensatory mechanisms in patients show a decreasing capacity to restore homeostasis, resulting in impaired resilience and neuroprogression (10, 11, 41).

However, the associations between biological parameters and characteristics of clinical course are rather small. It remains to be determined whether the differences in biological markers between patients and controls are inherent characteristics of the disease pathophysiology or rather a consequence of confounding factors such as psychopharmacological treatment, smoking status, or other lifestyle factors. Nearly all patients received psychopharmacological treatment that evidently affects monoamine metabolism. Differences in amino acid levels did not remain significant after adjustment for smoking status. Conflicting data on the effect of smoking on monoamine metabolism (42–44) and the high proportion of smokers in our patient group vs. the low proportion in controls make the interpretation of these results difficult.

## Strengths and limitations

We included patients in manic, depressive, and mixed episodes and completed approximately 6 test moments during a follow-up of 8 months. The longitudinal design enables a within-person assessment of diverse mood states and the high number of assessments by mood state increases the power of the study. The impact of methodological bias was minimized by standardized blood sampling and uniform, meticulous laboratory procedures. All clinical assessments were done by the same clinician-researcher, excluding interrater bias. Data on illness course and medication use were collected carefully. We used robust, transparent statistical methods. Mixed model analysis enables correction for missed moments, drop-out and a random variation in time and subject. The statistical models were adjusted for possible influences of BMI, smoking status, age, and albumin levels. The naturalistic design has several inherent limitations. Despite strict in- and exclusion criteria, the patient sample remained heterogeneous regarding characteristics as illness severity, duration of illness, treatment history, diagnosis, and history of substance abuse. Sample heterogeneity may hide relevant information that could have been discerned in a more homogenous patient group. We carefully collected data regarding course of illness and patient characteristics and integrated these data in the statistical analysis. Apart from the use of anti-inflammatory medication and ECT, there were no treatment restrictions during follow-up. Since patients were included during an acute mood episode, nearly all had changes in psychopharmacological treatment.

## Conclusion

We found stronger correlations between pro-inflammatory markers and cytotoxic pathways of monoamine metabolism in patients vs. controls. Middle-aged patients and patients with longer duration of illness had increased inflammatory and cytotoxic markers compared to young patients and controls. A pro-inflammatory proneness of patients and a subsequent shift of monoamine metabolism toward more cytotoxic pathways could underlie neuroprogression in BD. Since only few associations are found between biological markers and characteristics of clinical course, it remains to be determined if alterations in biological markers are due to a disease effect or rather a consequence of confounding factors.

## Author contributions

MM, VC, and SvdA developed the study protocol. SvdA did the patient recruitment, screening, and clinical assessments and first drafted the manuscript. DF did the laboratory analyses and supervised the data interpretation. Statistical analyses were done by SvdA. All authors contributed to the development of the manuscript and have approved the final version of the manuscript.

### Conflict of interest statement

SvdA was supported by a grant from Janssen Research and Development, a division of Janssen Pharmaceutica N.V. PdB and MT are employees of Janssen Pharmaceutica N.V. MM received grants and personal honoraria from Janssen Pharmaceutica N.V., AstraZeneca, Lundbeck, Bristol-Myer Squibb, and Eli Lilly. The remaining authors declare that the research was conducted in the absence of any commercial or financial relationships that could be construed as a potential conflict of interest.
