# Supplementary material for: Markers of Inflammation and Monoamine Metabolism Indicate Accelerated Aging in Bipolar Disorder
Source: Front Psychiatry. 2018 Jun 14;9:250. doi: 10.3389/fpsyt.2018.00250 (PMC6010913; doi:10.3389/fpsyt.2018.00250)
Supplement: Supplementary file 1 [file Table_1.DOCX]

**Supplementary material: Table S1-S4**

| **Table S1: Symptom severity by mood state in patients (baseline and all test moments combined)** | | | | |
| --- | --- | --- | --- | --- |
|  | **Euthymia** | **Depression** | **(Hypo)mania** | **Mixed episode** |
| **Baseline** | | | | |
| N |  | 29 | 29 | 9 |
| HDRS, mean (SD) |  | 21.2 (3.8) | 6.8 (4.2) | 21.0 (4.6) |
| YMRS, mean (SD) |  | 3.8 (3.3) | 20.9 (5.3) | 15.8 (4.7) |
| PANSS pos, mean (SD) |  | 8.9 (2.8) | 14.7 (4.6) | 15.4 (4.3) |
| PANSS neg, mean (SD) |  | 11.2 (4.9) | 8.3 (3.1) | 8.9 (2.4) |
| Psychotic features, N (%) |  | 3 (17.7) | 10 (58.8) | 4 (23.5) |
| **All test moments (baseline + follow-up)** | | | | |
| N | 64 | 178 | 71 | 23 |
| HDRS, mean (SE) | 3.9 (2.2) | 16.6 (5.8) | 6.6 (4.1) | 20.5 (4.4) |
| YMRS, mean (SE) | 2.7 (2.4) | 3.5 (2.7) | 18.3 (7.0) | 15.2 (4.4) |
| PANSS pos, mean (SE) | 7.4 (0.8) | 8.6 (2.3) | 13.5 (4.7) | 14.6 (4.2) |
| PANSS neg, mean (SE) | 7.6 (1.3) | 10.4 (4.0) | 8.3 (2.4) | 9.2 (2.4) |
| Psychotic features, N (%) | 0 | 16 (9.0) | 21 (29.6) | 7 (30.4) |

HDRS: Hamilton depression rating scale; YMRS: Young mania rating scale; PANSS: positive and negative symptom scale

| **Table S2: Levels of markers of inflammation and monoamine metabolism in patients and controls** | | | |
| --- | --- | --- | --- |
|  | Patients | Controls |  |
| IFN-y (pg/ml)^*^ | 4.46 (0.07) | 4.87 (0.10) | F(92.7)=0.5; p=0.465; b=0.09 |
| IL-6 (pg/ml)^*^ | 0.50 (0.06) | 0.41 (0.09) | F(96.2)=2.6; p=0.111; b=0.18 |
| TNF-α (pg/ml)^*^ | 1.91 (0.04) | 1.77 (0.05) | F(99.3)=1.4; p=0.237; b=0.08 |
| CRP (mg/L)^*^ | 1.92 (0.13) | 1.27 (0.17) | F(100.9)=3.7; p=0.056; b=0.41 |
| Trp (μmol/l) | 50.99 (0.87) | 54.36 (1.20) | F(92.4)=5.2; p=**0.026**; b=3.37 |
| Kyn (μmol/l) | 1.46 (0.04) | 1.58 (0.05) | F(97.6)=3.6; p=0.061; b=0.12 |
| Kyn/Trp (μmol/mmol) | 29.06 (0.66) | 29.41 (0.91) | F(99.3)=0.1; p=0.756; b=0.35 |
| Neo (nmol/l) | 5.07 (0.13) | 5.04 (0.18) | F(99.6)=0.0; p=0.910; b=0.02 |
| Tyr (μmol/l) | 65.48 (2.01) | 75.05 (2.77) | F(98.2)=7.8; p=**0.006**; b=9.56 |
| Phe (μmol/l) | 50.78 (0.98) | 55.93 (1.34) | F(95.0)=9.6; p=**0.003**; b=5.14 |
| Phe/Tyr | 0.81 (0.02) | 0.78 (0.02) | F(99.4)=1.3; p=0.259; b=0.03 |

Data presented as mean (SE).

IFN-y: interferon gamma; IL: interleukin; TNF-α: tumor necrosis factor alpha; CRP: C-reactive protein; Trp: tryptophan; Kyn; kynurenine; Neo: neopterin; Tyr: tyrosine; Phe: phenylalanine.

*SE on log-transformed data

| **Table S3: Correlations among biological markers in patients (lower left) and *controls (upper right*), spearman’s ρ + significance levels** | | | | | | | | | | | |
| --- | --- | --- | --- | --- | --- | --- | --- | --- | --- | --- | --- |
|  | **Trp** | **Kyn** | **Kyn/Trp** | **Neo** | **Tyr** | **Phe** | **Phe/Tyr** | **IFN-y** | **IL-6** | **TNF-α** | **CRP** |
| **Trp** | 1 | *0,39^***^* | *-0,29^***^* | *-0,08* | *0,45^***^* | *0,48^***^* | *-0,23^**^* | *-0,04* | *-0,09* | *-0,00* | *-0,20^**^* |
| **Kyn** | 0,43^***^ | 1 | *0,72^***^* | *0,19^**^* | *0,27^***^* | *0,26^***^* | *-0,20^**^* | *0,31^***^* | *0,08* | *0,23^**^* | *-0,01* |
| **Kyn/Trp** | -0,30^***^ | 0,68^***^ | 1 | *0,27^***^* | *0,00* | *-0,05* | *-0,07* | *0,34^***^* | *0,15* | *0,27^*^* | *0,1* |
| **Neo** | -0,07 | 0,37^***^ | 0,43^***^ | 1 | *-0,02* | *-0,10* | *-0,07* | *0,35^***^* | *0,28^**^* | *0,26^***^* | *0,25^***^* |
| **Tyr** | 0,38^***^ | 0,22^***^ | -0,04 | -0,15^**^ | 1 | *0,72^***^* | *-0,79^***^* | *0,06* | *-0,07* | *-0,00* | *-0,07* |
| **Phe** | 0,40^***^ | 0,19^***^ | -0,10 | -0,07 | 0,69^***^ | 1 | *-0,21^**^* | *-0,02* | *-0,16^*^* | *-0,06* | *-0,14* |
| **Phe/Tyr** | -0,17^**^ | -0,13^*^ | -0,02 | 0,17^**^ | -0,73^***^ | -0,06 | 1 | *-0,11* | *-0,06* | *-0,04* | *-0,05* |
| **IFN-y** | -0,07 | 0,12 | 0,17^**^ | 0,31^***^ | -0,09 | -0,06 | 0,05 | 1 | *0,39^***^* | *0,51^***^* | *0,22^**^* |
| **IL-6** | -0,18^**^ | 0,04 | 0,19^**^ | 0,20^**^ | 0,06 | -0,05 | -0,14^*^ | 0,33^***^ | 1 | *0,50^***^* | *0,48^***^* |
| **TNF-α** | 0,04 | 0,33^***^ | 0,32^***^ | 0,33^***^ | -0,06 | -0,06 | 0,04 | 0,43^***^ | 0,47^***^ | 1 | *0,17^*^* |
| **CRP** | -0,08 | -0,03 | -0,01 | 0,23^***^ | 0,05 | 0,02 | -0,04 | 0,17^**^ | 0,48^***^ | 0,03 | 1 |

**p*<0.05; ***p*<0.01; ****p*<0.001

Trp: tryptophan; Kyn; kynurenine; Neo: neopterin; Tyr: tyrosine; Phe: phenylalanine; IFN-y: interferon gamma; IL: interleukin; TNF-α: tumor necrosis factor alpha; CRP: C-reactive protein.

| **Table S4: Correlations of biological markers to symptom severity, spearman’s ρ + significance levels** | | | |
| --- | --- | --- | --- |
|  | HDRS | YMRS | PANSS pos |
| Trp | -0.13^*^ | 0.17^**^ | 0.15^**^ |
| Kyn | -0.14^*^ | 0.17^**^ | 0.11^*^ |
| Kyn/Trp | -0.05 | 0.03 | -0.01 |
| Neo | 0.02 | -0.01 | -0.01 |
| Tyr | -0.11^*^ | 0.21^***^ | 0.21^***^ |
| Phe | -0.03 | 0.18^**^ | 0.22^***^ |
| Phe/Tyr | 0.13^*^ | -0.10 | -0.07 |
| IFN-y | -0.11 | 0.02 | -0.00 |
| IL-6 | -0.08 | 0.04 | 0.00 |
| TNF-α | -0.02 | 0.08 | 0.08 |
| CRP | 0.02 | 0.05 | 0.03 |

**p*<0.05; ***p*<0.01; ****p*<0.001
